# TMS in major depression: A retrospective naturalistic study including two subjective tools

**DOI:** 10.1177/10398562251314301

**Published:** 2025-01-20

**Authors:** Saxby Pridmore, Gregory M Peterson, Marzena Rybak, Karen Byrne, Tae Dillon, Yvonne Turnier-Shea, Ahmed Naguy

**Affiliations:** Discipline of Psychiatry, 3925 University of Tasmania, Hobart, TAS, Australia; School of Pharmacy and Pharmacology, 3925 University of Tasmania, Hobart, TAS, Australia; Hobart TMS, Bellerive, TAS, Australia; Hobart TMS, Bellerive, TAS, Australia; Hobart TMS, Bellerive, TAS, Australia; Hobart TMS, Bellerive, TAS, Australia; Al-Manara CAP Centre, KCMH, Shuwaikh, Kuwait

**Keywords:** transcranial magnetic stimulation, major depressive disorder, mood assessment tools, remission, prediction

## Abstract

**Objective:**

To report the outcomes of transcranial magnetic stimulation (TMS) treatment of patients with acute major depressive disorder (MDD), with particular attention to the performance of the individual assessment tools, including two new subjective mood scales.

**Methods:**

Patients with MDD were treated with up to 35 daily TMS sessions. Objective quantification of mood utilised the Hamilton Depression Rating Scale (HAM-D6) and the Clinical Global Impression-Severity scale (CGI-S). Subjective quantification was made using the Subjective Depression Scale (SDS6) and a new Daily Emotion Score (DES) – a single question which is asked daily.

**Results:**

Ninety consecutive patients (58 females; 64.4%) with a mean age of 46.9 years were included. Using HAM-D6 criteria, 51 patients (56.7%) achieved remission. Scores obtained using the different tools correlated well at the same time point, especially at the conclusion of TMS therapy. The only statistically significant independent predictors of remission were the percentage improvement at session 10 (relative to baseline) in the SDS6 (*p* = .0026) and in the DES (*p* = .043).

**Conclusion:**

The SDS6 was confirmed as a valuable companion for the HAM-D6. The DES correlated with the other subjective tool (SDS6); the latter, in particular, may also have utility in predicting treatment outcome.

Transcranial magnetic stimulation (TMS) is a safe and effective treatment of acute major depressive disorder (MDD).^1^ The length of a course of treatment varies from one service to another, typically from 20 to 35 single daily (5 days per week) treatments. Recent evidence suggests that the response to TMS may be most accurately assessed through the use of multiple instruments^2^; in that study, four scales were used, two clinician-rated and two patient-rated scales. It was concluded there is a need for additional research examining multiple rating instruments in routine TMS treatment, especially ones that assess different symptom domains.

Our group also uses four assessment scales, including the clinician observer-rated (objective) six-item Hamilton Depression Rating Scale (HAM-D6^3^) and the Clinical Global Impression-Severity scale (CGI-S^4^). We also use two patient self-rated (subjective) tools which we developed ourselves. The six-item Subjective Depression Scale (SDS6) is a companion for the HAM-D6.^5^ Evidence indicates it correlates with HAM-D6 scores.^6^ The Daily Emotion Score (DES) is described in a case study^7^ but has been awaiting scientific validation. It is a single question which is asked and answered each treatment day: ‘On a scale of 1 to 10, how are you feeling today? One is very low indeed, and 10 means not even slightly sad or depressed’. Most mood scale scores decrease as the mood improves – in contrast, the DES increases as mood improves.

In addition to mental disorders, factors which impact DESs include personality and life circumstances. In the DSM5 diagnostic criteria of MDD, ‘depressed mood’ and ‘diminished interest or pleasure’ (along with others) are present ‘nearly every day’.^8^ The presence of such symptoms lowers the DES – however, they may not be present *every* day; thus, there may be periods when the subjective experience of the disorder is less distressing. Personality differs across individuals; some people report greater daily variation in DESs than others. The circumstances of individuals’ lives change – for instance, winning a lottery or the arrival of a granddaughter – can increase DESs. Generally, MDD anchors DESs at low levels. As the MDD remits, the DESs gradually rise; concurrent personality features and altered life circumstances can trigger unexpected variations, which may prompt discussion.

Researchers have observed conventional TMS in acute MDD as series of 20 to 35 treatments and commented on response trajectories. For instance, Fitzgerald et al.^9^ reported that improvement of active cases (compared to sham) was apparent at 2 weeks and continued across a 6-week period. Kaster et al.^10^ identified four trajectories: rapid response, no response, and two intermediate trajectories. They reported the rapid-responding group achieved great improvement by week 2. Hsu et al.^11^ conducted a meta-analysis of 40 studies and found the ‘peak effectiveness’ occurred in the first 3 weeks.

Some evidence suggests a positive response by session 10 predicts a better long-term outcome.^2,12^ However, others caution that a poor early response does not guarantee a poor final response, and early withdrawal may be unwise.^13^ Supporting this, ‘late responders’ have been identified who have responded after courses up to 72 treatments.^14,15^ It is also widely stated that the more severe the depression at commencement, the less the chances of achieving remission with TMS.^9,10^

Our aim was to examine the response of acute MDD to a course of TMS and to pay particular attention to the performance of the assessment tools, including two new subjective mood scales. In addition, we wished to examine the relationship between early response and treatment outcome.

## Methods

This clinical audit of routine treatment was conducted at a private TMS outpatient service. When patients commence treatment, they are fully informed and sign an agreement allowing their anonymised data to be pooled, analysed, and potentially reported in the medical literature. The St. Helens Private Hospital (Hobart, Tasmania, Australia) Medical Advisory Committee approved the study and deemed it did not need formal ethics approval.

Patients experiencing acute MDD who had failed to respond to adequate courses of at least two different antidepressants were offered treatment. TMS was delivered to the left dorsolateral prefrontal cortex (DLPFC), 10 Hz stimulation, 120% resting motor threshold, 4 second trains, and 75 trains per treatment (3000 pulses per treatment), delivered with a MagPro R30 device (MagVenture; Lucernemarken 15, DK-3520 Farum, Denmark). The DLPFC was located 5.5 cm anterior to the point at which the resting motor threshold was determined. Up to 35 daily treatments were available. No changes in medication were made over the TMS treatment period.

To objectively quantify mood, (i) the HAM-D6 (the primary measure) was administered on the first and final day of treatment (<4 indicates ‘remission’^16^) and (ii) the CGI-S was administered on days 1, 10, and 20 and the final day of treatment (<2 indicates remission^17^). To subjectively quantify mood, (i) the SDS6 was administered on days 1, 10, and 20 and the final day of treatment and (ii) the DES was administered on each treatment day.

Differences in baseline demographics and clinical assessment tool scores between those patients who did, and did not, achieve remission (HAM-D6 ≤ 4) at the end of the course of TMS were examined using t-tests and chi-square tests for numerical and categorical variables, respectively. Correlations between scores with the different assessment tools were examined using the Pearson correlation coefficient. Independent associations between variables and clinical response (remission/non-remission) were examined using multiple logistic regression. A *p* value below 0.05 (two-tailed) was considered statistically significant for all tests. The analyses were performed using SPSS (IBM Corp., IBM SPSS Statistics for Windows, version 27, 2020, Armonk, NY, USA).

## Results

**Figure 1.**
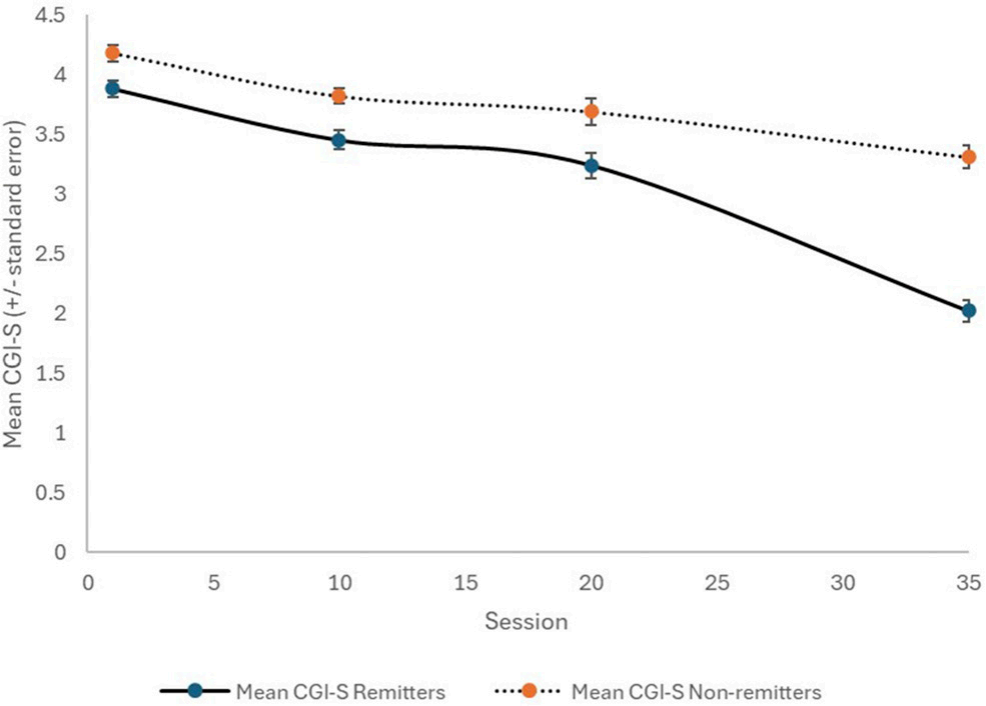
Change in CGI-S with TMS therapy, for remitters (solid line) and non-remitters (dotted line).

**Figure 2.**
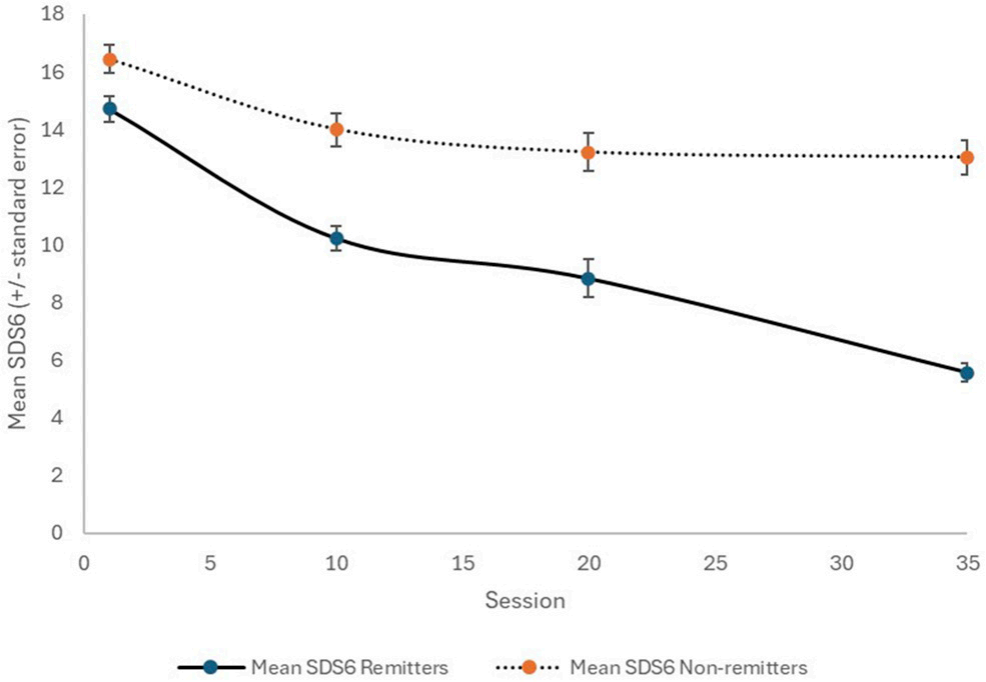
Change in SDS6 with TMS therapy, for remitters (solid line) and non-remitters (dotted line).

**Figure 3.**
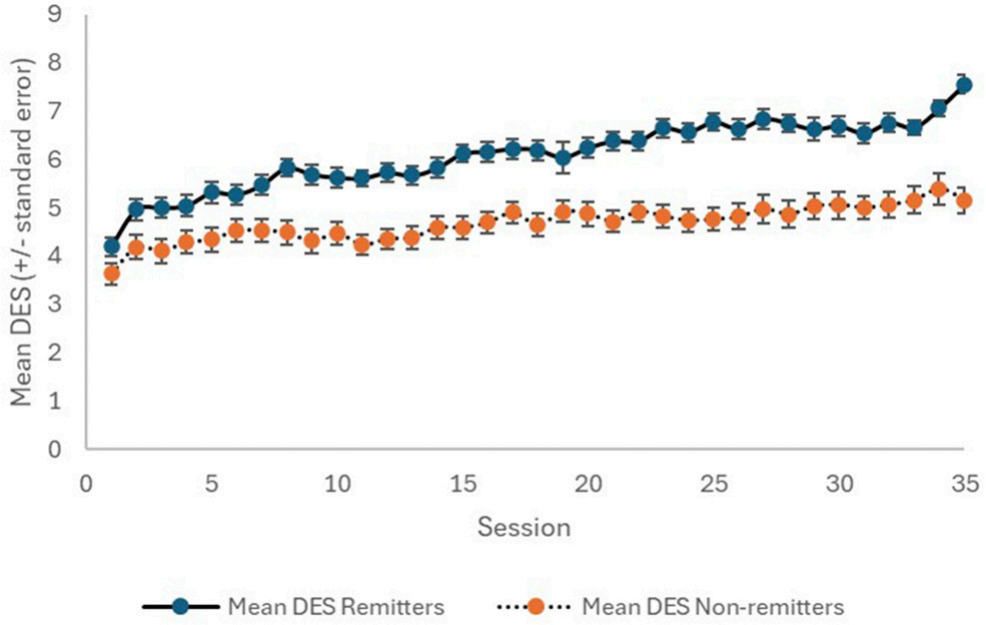
Change in DES with TMS therapy, for remitters (solid line) and non-remitters (dotted line).

Ninety consecutive patients (58 females; 64.4%) with a mean age of 46.9 years were included (Table 1). Using HAM-D6 criteria, 51 of the patients (56.7%) achieved remission. The patients who achieved HAM-D6 remission reached a final CGI-S mean score of 2.0 (compared to 3.3 for those who did not achieve remission; *p* < .0001), which is in the CGI-S remission zone. The mean SDS6 and DESs at the conclusion of treatment were also markedly different (*p* < .0001) between the ‘remitters’ and ‘non-remitters’. The temporal changes with treatment for the CGI-S, SDS6, and DES are shown in Figures 1–3, respectively. Interestingly, the clear difference between remitters and non-remitters seems evident by session 10 for the self-rated SDS6 and DES, while their divergence appears later with the clinician-rated CGI-S.

**Table 1.** Study sample characteristics.

	Total sample (*n* = 90)	Remitters (*n* = 51)	Non-remitters (*n* = 39)
Age in years (SD)	46.9 (14.3)	45.6 (14.2)	48.5 (14.5)
Females (%)	58 (64.4)	35 (68.6)	23 (59.0)
Baseline HAM-D6 [mean (SD)]	8.1 (1.8)	7.7 (1.6)	8.6 (1.8)^a^
Final HAM-D6 [mean (SD)]	4.8 (2.4)	3.1 (1.1)	6.9 (1.9)^b^
Baseline CGI-S [mean (SD)]	4.0 (0.5)	3.9 (0.5)	4.2 (0.5)^c^
Session 10 CGI-S [mean (SD)]	3.6 (0.5)	3.5 (0.6)	3.8 (0.4)^c^
Final CGI-S [mean (SD)]	2.6 (0.9)	2.0 (0.6)	3.3 (0.6)^b^
Baseline SDS6 [mean (SD)]	15.5 (3.2)	14.7 (3.1)	16.4 (3.0)^c^
Session 10 SDS6 [mean (SD)]	11.9 (3.7)	10.2 (3.0)	14.0 (3.5)^b^
Final SDS6 [mean (SD)]	8.8 (4.8)	5.6 (2.3)	13.0 (3.8)^b^
Baseline DES [mean (SD)]	4.0 (1.4)	4.2 (1.4)	3.6 (1.3)^a^
Session 10 DES [mean (SD)]	5.1 (1.6)	5.6 (1.4)	4.5 (1.5)^c^
Final DES [mean (SD)]	6.5 (1.6)	7.6 (1.0)	5.2 (1.2)^b^

^a^*p* < .05 for difference between remitters and non-remitters.

^c^*p* < .01 for difference between remitters and non-remitters.

^b^*p* < .0001 for difference between remitters and non-remitters.

There was no significant difference between remitters and non-remitters with regard to either sex or age (Table 1). The non-remitters had significantly worse baseline mean scores for each of the measurement tools (HAM-D6, CGI-S, SDS6, and DES). Furthermore, the 10-day measurements (CGI-S, SDS6, and DES) were also significantly worse for the non-remitters.

The correlation between each of the measurement tools at the commencement and conclusion of TMS therapy is shown in Table 2. Of note, the HAM-D6 and SDS6 were highly correlated, particularly at the conclusion of treatment (r = 0.92). The SDS6 scores were almost exactly double the HAM-D6 scores. The DES at treatment conclusion was highly correlated with the HAM-D6, CGI-S, and SDS6 at the same time point, but not at baseline. Similarly, the SDS6 became more strongly correlated with the other measures over time.

**Table 2.** Correlation matrix for the depression rating instruments.

	Baseline HAM-D6	Final HAM-D6	Baseline CGI-S	Final CGI-S	Baseline SDS6	Final SDS6	Baseline DES	Final DES
Baseline HAM-D6	1	0.33	0.425	0.337	0.712	0.397	−0.315	−0.392
Final HAM-D6	0.33	1	0.559	0.881	0.393	0.919	−0.251	−0.796
Baseline CGI-S	0.425	0.559	1	0.607	0.386	0.601	−0.256	−0.487
Final CGI-S	0.337	0.881	0.607	1	0.365	0.85	−0.101	−0.814
Baseline SDS6	0.712	0.393	0.386	0.365	1	0.475	−0.324	−0.468
Final SDS6	0.397	0.919	0.601	0.85	0.475	1	−0.159	−0.824
Baseline DES	−0.315	−0.251	−0.256	−0.101	−0.324	−0.159	1	0.133
Final DES	−0.392	−0.796	−0.487	−0.814	−0.468	−0.824	0.133	1

While, as mentioned above, the non-remitters had significantly worse baseline mean scores for each of the measurement tools, a multiple logistic regression analysis incorporating age, sex, and the baseline measures for each of HAM-D6, CGI-S, SDS6, and DES found no statistically significant independent predictors of response (remission/non-remission). We performed a second multiple logistic regression analysis incorporating age, sex, the baseline measures for each of HAM-D6, CGI-S, SDS6, and DES, and the percentage improvements at session 10 (relative to baseline) for each of CGI-S, SDS6, and DES. It was found that the only statistically significant independent predictors of remission were the percentage improvement at session 10 in the SDS6 (*p* = .0026) and the percentage improvement at session 10 in the DES (*p* = .043). The mean improvement in SDS6 at session 10 in the remitters was 29.8% (SD: 21.3) compared to 13.0% (SD: 23.2) in the non-remitters. The corresponding values for the DES were 59.2% (SD: 105.3) and 36.9% (SD: 66.4) for remitters and non-remitters, respectively.

## Discussion

Careful blind placebo-controlled studies have proven TMS is an effective treatment of MDD.^9^ In this naturalistic study, we found a remission rate of 57%, which is in the range of remission results (28%–62%) reported in similar observational studies.^18^

In assessing response to treatment, it is sensible and recommended^2^ that both the objective and subjective aspects of MDD be considered. The HAM-D6 is a long-established objective tool, which has the advantage of being brief and time efficient. We developed the SDS6 as a companion for the HAM-D6 and previously presented some supportive material.^6^ The current study provides additional strong evidence that the SDS6 complements HAM-D6 results. Figure 2 and our statistical analysis also suggest that the SDS6 can change relatively quickly with TMS therapy, and the extent of early change may indicate the likelihood of achieving remission by the end of a course of TMS.

It is of interest that, while the scoring options are the same (0–4 for 5 items and 0–2 for 1 item), the total scores of the SDS6 were usually double those of the HAM-D6. This apparently reflects the difference between the observer’s observations of the patient and the patient’s personal experience of the disorder.

The DES is a unique tool – a single question about the emotional state of the individual, which is administered daily. It is unique and interesting. In practice, unexpected falls or rises in the DES often trigger patient–staff discussions and may lead to the revelation of new clinically relevant information. We propose it is influenced by the degree of depressive disorder and other factors including personality and life circumstances. We have shown the DES correlates with the HAM-D6 and SDS6, especially over time, and thus confirmed it as a valuable assessment tool. To a lesser extent than the SDS6, the magnitude of early change in the DES may also indicate the likelihood of achieving remission by the end of a course of TMS.

Limitations of the study should be acknowledged. It was a retrospective naturalistic study, not a randomised, controlled trial. It is possible that the observed improvements could be due to placebo effects or natural fluctuations in the course of MDD. We also did not formally assess patient functioning or quality of life, and examined a relatively limited range of variables when attempting to predict treatment response. In particular, we did not have a 10-day measurement for the HAM-D6. Importantly, neither the SDS6 nor the DES has undergone formal scientific validation.

## Conclusion

The study confirms that TMS is an effective treatment for resistant MDD, and that the assessment tools were mutually supportive. The subjective SDS6 proved a valuable companion for the objective HAM-D6, and the magnitude of its early changes may be a useful indicator of likelihood of response to TMS, while the DES deserves further study.
